# A Global Model for the Estimation of Speeds of Sound in Deep Eutectic Solvents

**DOI:** 10.3390/molecules25071626

**Published:** 2020-04-01

**Authors:** Hamed Peyrovedin, Reza Haghbakhsh, Ana Rita C. Duarte, Sona Raeissi

**Affiliations:** 1 School of Chemical and Petroleum Engineering, Shiraz University, Mollasadra Ave., Shiraz 71348-51154, Iran; hamed.peyro@yahoo.com (H.P.); r.haghbakhsh@shirazu.ac.ir (R.H.); 2 LAQV, REQUIMTE, Departamento de Química da Faculdade de Ciências e Tecnologia, Universidade Nova de Lisboa, 2829-516 Caparica, Portugal; ard08968@fct.unl.pt

**Keywords:** Deep Eutectic Systems, green solvent, physical property, sound velocity, correlation, modeling

## Abstract

Deep eutectic solvents (DESs) are newly introduced green solvents that have attracted much attention regarding fundamentals and applications. Of the problems along the way of replacing a common solvent by a DES, is the lack of information on the thermophysical properties of DESs. This is even more accentuated by considering the dramatically growing number of DESs, being made by the combination of vast numbers of the constituting substances, and at their various molar ratios. The speed of sound is among the properties that can be used to estimate other important thermodynamic properties. In this work, a global and accurate model is proposed and used to estimate the speed of sound in 39 different DESs. This is the first general speed of sound model for DESs. The model does not require any thermodynamic properties other than the critical properties of the DESs, which are themselves calculated by group contribution methods, and in doing so, make the proposed method entirely independent of any experimental data as input. The results indicated that the average absolute relative deviation percentages (AARD%) of this model for 420 experimental data is only 5.4%. Accordingly, based on the achieved results, the proposed model can be used to predict the speeds of sound of DESs.

## 1. Introduction

In recent years, various studies have been published regarding the negative impacts of volatile organic solvents on our planet. Such studies put forth the concerns regarding the use of such harmful compounds, and consequently, encouraged researchers to introduce novel green solvents as environmentally friendly replacements for the commonly used polluting substances [1,2]. In this respect, different types of green substances were introduced, for example, the ionic liquids (ILs). ILs have certain advantages, such as low vapor pressures and insignificant volatilities, tunable properties, chemical and thermal stabilities and acceptable solvent power [3,4]. Such characteristics have turned them into an interesting family of green solvents for research. Consequently, their applications have been investigated in various fields, for example carbon capture, separation operations, chemical synthesis, catalysis, biodiesel production and as sustainable lubricants [5,6,7,8,9]. However, with time, some disadvantages have also been reported for ILs, such as their high price, the need for multiple-step purification and, in some cases, toxicity [3,6,10]. These issues have caused some limitations in their applications. Accordingly, it is worthwhile to propose new types of green solvents to overcome the limitations of ILs.

Recently, Abbott et al. suggested a new family of solvents that can be prepared simply by the mixing of two substances [11,12]. These two components are a hydrogen bond acceptor (HBA) and a hydrogen bond donor (HBD). When the HBA and HBD are mixed together, a mixture is formed with a melting point that is much lower than those of the individual HBA and HBD [13,14,15,16]. Due to this, these novel solvents are named the deep eutectic solvents (DESs). DESs have nearly all of the advantages of ILs, while they have the added benefits of easy preparation by the simple mixing of the HBA and HBD, nontoxicity (for most DESs), biodegradability and biocompatibility [17,18]. Moreover, a comparison between the costs of ILs and DESs shows that DESs are generally cheaper than ILs [17]. Accordingly, these novel solvents have attracted great attention due to their unique characteristics. Based on the desirable properties and advantages of DESs, some studies have suggested that DESs have the potential to be used for various applications in the industries, such as in extraction and separation processes, chromatography, biodiesel production, drug delivery systems and for introducing novel drugs [9,12,15,17,19,20]. Furthermore, DESs are designer solvents, i.e., suitable DESs possessing the desired (thermo-)physical properties can be designed by the engineered choice of the HBA, HBD and their molar ratios. In being so, a large number of DESs can be prepared [18]. Therefore, research on DESs is steeply on the rise, and the rate at which basic knowledge will become available on the physical properties of DESs will probably lag behind the introduction of the numerous upcoming DESs. This problem is actually an obstacle to the industrial use of DESs [21,22].

The speed of sound is an important thermodynamic property, which can be used to determine various other properties, such as density, heat capacity, the Joule–Thomson coefficient, bulk modulus, virial coefficients and equation of state constants [23,24]. This characteristic has made the speed of sound a noteworthy property. This is even more pronounced for the particular family of DESs, which have even greater shortages of property data than the conventional solvents. Some of the significant thermophysical properties of DESs that are lacking can be calculated using the speed of sound. Among the most important process design and optimization properties, one can point to for example, the isentropic and isothermal compressibilities, heat capacities, and thermal conductivities of DESs. This issue is highlighted when considering that DESs are designer solvents, for which most of the thermophysical properties of the newly introduced and upcoming DESs are unknown [25,26].

Accordingly, based on its value in predicting other unknown properties, different studies have already been published to estimate the speeds of sound of the older generation of designer solvents, i.e., the ionic liquids. Gardas and Coutinho [27], presented the following relation to calculate the speed of sound, *u*, in ILs:

(1) $$\log\left(u\right)=\alpha \log\left(\frac{\sigma }{\rho }\right)+\beta$$

where *ρ* and *σ* are the density and the surface tension, respectively, and *α* and *β* are the correlation’s optimized constants [27].

In other published work, Hekayati and Esmaeilzadeh [28] introduced a correlation to predict the speed of sound of ILs. In their model, the speed of sound can be calculated as:

(2) $$\ln\left(u\right)=\left(a+bM_{W}\right)\ln\left(\frac{\sigma }{\rho }\right)+cM_{W}+dM_{W}^{2}-eT+f$$

where *M_w_*, *ρ*, *σ* and *T* are the molecular weight, density, surface tension and temperature, respectively, and the constants *a* to *f* are optimized parameters [28]. Based on Equation (2), Hekayati and Esmaeilzadeh calculated the speed of sound of 48 different ILs and showed that their model can estimate this property with good accuracy (1.11% and 1.62% for the training ant test datasets, respectively).

In 2010, Singh and Singh [29] optimized the adjustable parameters *ψ* and *ξ*, and used Equation (3) for calculating the speed of sound in ILs.

(3) $$\log\left(u\right)=\psi \log\left(\frac{\sigma }{\rho }\right)+\xi$$

In all of these three literature models, the density and surface tension of the IL must be known to calculate the speed of sound. When such physical properties are unavailable, Haghbakhsh and his coworkers introduced a method in which the speed of sound can be calculated using an atomic contribution model [24]:

(4) $$u=\frac{A}{M_{w}}+BT$$

(5) $$A=\overset{n}{\underset{i=1}{\sum }}n_{i}\times \Delta A_{i}$$

(6) $$B=\overset{n}{\underset{i=1}{\sum }}n_{i}\times \Delta B_{i}$$

In this model, Δ*A_i_* and Δ*B_i_* are the optimized parameters and *n_i_* is the number of atoms of type *i* in the molecule.

All of the above methods were specific to ionic liquids, and up to date, there are no general models available for estimating the speeds of sound in DESs. So far, only experimental data is available on the speeds of sound of DESs. The aim of this work was to introduce, for the first time, an accurate, simple and easy-to-use generalized model for estimating the speed of sound in various DESs. To have wide applicability, the idea was to propose a model, which does not require experimental physical property data as its input.

## 2. Methods

### 2.1. Selected Deep Eutectic Solvents (DESs) and Experimental Data

In this work, 420 speed of sound data were collected from literature references, covering 39 different DESs [21,30,31,32,33,34,35,36,37,38,39,40,41,42]. Table 1 lists these 39 DESs, including their corresponding HBAs, HBDs and molar ratios.

The collected data points were divided randomly into training and test groups. The training dataset, consisting of 292 data (69%) and 28 DESs, was used for developing the model, while the test group, which consists of 128 data (31%) and 11 DESs, was later used to determine the accuracy of the proposed model.

### 2.2. The Model

One of the main objectives of this work was to propose a widely applicable method. For this purpose, not only the most up-to-date and complete dataset available was used for generality, but also, attention was given to the choice of input parameters. It was the goal of this work to propose a model to be applicable to even those DESs that have not yet been prepared in the laboratories. This is of great significance by considering that, similar to ILs, DESs are designer solvents. Accordingly, a huge number of DESs are possible, and numerous new DESs will appear in future research. Due to this, it would be most desirable to predict the property of a DES, and the feasibility of its utilization in a particular task, before actually undergoing any experimental expense and time. With this idea in mind, we attempted to develop a model in which the only required input data were the critical properties and acentric factor of the DESs, which can themselves be calculated by group contrition methods. In this way, the only information necessary is practically the molecular structures of the HBA and HBD.

Therefore, the aforementioned properties were selected as the input parameters and various functionalities were investigated with the aid of genetic algorithm (GA) [43], as an optimization tool. Genetic algorithm is actually an approach, which is initiated by a set of random solutions, whereupon by iteratively applying a variety of stochastic operators to the solutions, they become successively evolved. This procedure is repeated until the final solutions satisfy a minimizing condition, which is defined by an operator as an objective function. The following objective function (OF) was utilized to optimize the parameters of the function, which relates the input parameters (the critical parameters and the acentric factor) to the target parameter (the speed of sound),

(7) $$OF=\frac{100}{N}\overset{N}{\underset{i}{\sum }}\left|\frac{u_{i,exp}-u_{i,cal}}{u_{i,exp}}\right|$$

In Equation (7), *u_i,exp_* and *u_i,cal_* are the experimental and calculated speed of sound, and *N* is the number of literature data. In this manner, different possible mathematical formulations were analyzed and tested to develop the generalized model for estimating the speeds of sound in DESs.

## 3. Results and Discussion

The modified Lydersen–Joback–Reid method [44,45] and the Lee–Kesler mixing rules [46] were used to calculate the acentric factor, critical temperature, critical pressure and critical volume of all 39 DESs [22,47]. The calculated values are presented in Table 2.

By considering the acentric factors and critical properties of the DESs presented in Table 2 as the input parameters, and by investigating various combinations of input parameters, a generalized model for estimating the speeds of sound in DESs is obtained, as Equation (8):

(8) $$u=\omega \left[7.378M_{w}-2.012T\right]-2.911V_{c}+2514.2$$

where *u*, *V_c_, M_w_* and *T* are the speed of sound, critical molar volume, molecular weight and the desired temperature in m/s, cm^3^/mol, g/mol and kelvins, respectively, and *ω* is the acentric factor.

**Figure 1:**
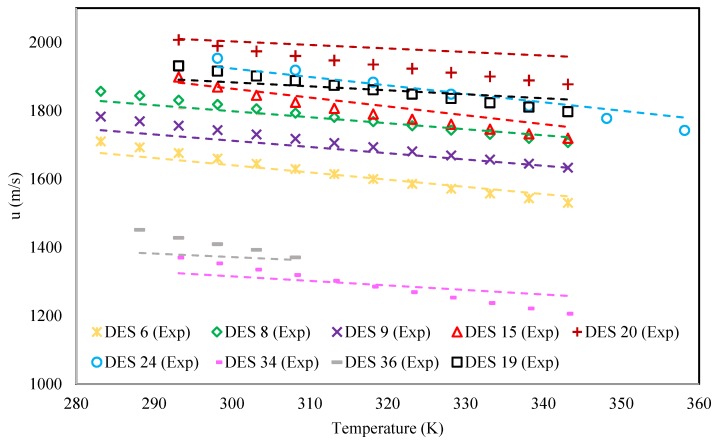
Comparison of the behavior of the speed of sound versus temperature for the proposed model and experimental data for nine randomly selected DESs.

In Figure 1, a comparison between the calculated speeds of sound of nine different DESs and the corresponding literature data is shown. It can be seen that the proposed model could successfully calculate the speeds of sound of the different-natured DESs, having different families of HBAs and HBDs. Within the temperature range of Figure 1, it was observed that at a constant pressure, the speed of sound had an almost linear relation to temperature in DESs. This linearity of the experimental data was followed reliably by the proposed model, having the constant slope of −2.012*ω* for each DES.

Following Equation (8), and using the available literature data, the average absolute relative deviation percentages (AARD%) of the proposed model for the training, test, and overall datasets were calculated using Equation (9)

(9) $$AARD\%=\frac{100}{N}\overset{N}{\underset{i}{\sum }}\left|\frac{u_{i,exp}-u_{i,cal}}{u_{i,exp}}\right|$$

According to the results presented in Table 3 for all three data sets, the calculated AARD% of all 420 data points was only 5.4%, which shows the accuracy and reliability of the proposed model. However, even more important was the AARD% of the test dataset, consisting of 128 data points, which was found to be 6.8%. Since all of the data in this dataset were unseen by the model (not used when developing the model), this shows the capability of the proposed model in predicting the speed of sound of new and upcoming DESs.

**Figure 2:**
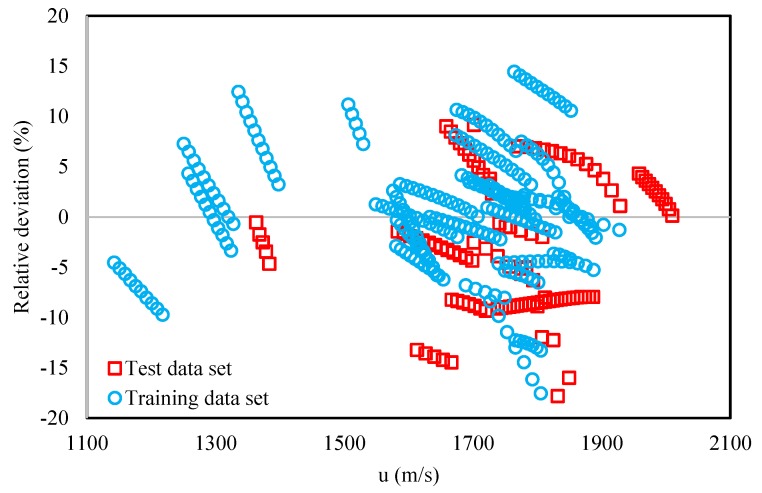
The relative deviation percent for the entire investigated data range for both the training and test datasets.

**Figure 3:**
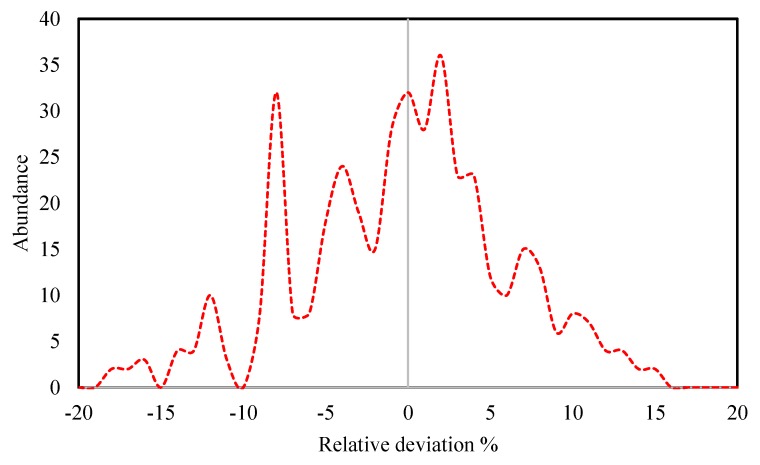
The distribution behavior of the relative deviation percent of the proposed model for the overall dataset.

While AARD% is a good indication of the average errors, it does not give any information on the over- and under-estimations of the model. Therefore, for further investigations, the relative deviation percentages (RD%) were also calculated using Equation (10) and presented in Figure 2; Figure 3.

(10) $$RD\%=100\left(\frac{u^{exp}-u^{cal}}{u^{exp}}\right)$$

where, *u^cal^* and *u^exp^* express the calculated and experimental speeds of sound, respectively.

In the calculation of RD%, both the test and training datasets were considered and the behavior of the relative deviation percent of these two datasets can be compared in Figure 2. Based on this figure, a rather normal behavior can be seen for both the test and training datasets, i.e., there are no systematic over- or under-estimations by the model for either of the two datasets. These observations not only provide the confidence of use of the model regarding any systematic errors, but also validate that apart from the correlative ability, the predictive use of the model is trustworthy. Furthermore, based on Figure 3, the distribution of the relative deviation percent is mostly concentrated close to zero. This further indicates the reliability of the proposed model. According to both Figure 2 and Figure 3, the maximum deviations of the proposed model with respect to experimental values were about ±20%. Such high error values occur for only a small number of the DESs, and again, are symmetric with respect to positive and negative deviations.

In order to compare the accuracy of the proposed model to published literature, the ionic liquid models of Gardas and Coutinho [27], Hekayati and Esmaeilzadeh [28], Singh and Singh [29] and Haghbakhsh et al. [24], which were presented as Equations (1)–(6), were considered as the next closest systems to DESs. In order to use Equations (1)–(3), the surface tensions and the densities of all of the DESs needed to be calculated. For this purpose, the densities of the DESs under study were calculated using the method proposed by Haghbakhsh et al. [22], and the surface tensions were calculated with the aid of Equation (11), proposed by Curl and Pitzer [48].

(11) $$\sigma =P_{c}^{2/3}T_{c}^{1/3}\frac{1.86+1.18\omega }{19.05}{\left[\frac{3.75+0.91\omega }{0.291-0.08\omega }\right]}^{2/3}{\left(1-T_{r}\right)}^{11/9}$$

The results of the calculated AARD% values for all the aforementioned methods are given in Table 4. According to the calculated AARD% values, the proposed correlation was the most accurate model among the compared literature models. In general, the proposed model decreased the AARD% values by almost 40% in comparison to the other three models. Of course, it must be emphasized that there were no literature models available specifically for DESs. All of the four literature models compared in Table 4 were proposed for ionic liquids, and not DESs, and so, one does not expect high accuracies when they are used to predict the speeds of sound in DESs. Apart from Singh and Singh’s model, which had very poor results for DESs, the other three models showed acceptable results, even though they were developed for ionic liquids.

Furthermore, the model of Haghbakhsh et al., similar to the model proposed in this study, does not require surface tension and density data of the DESs in order to calculate the speed of sound, which can be considered as an advantage of these two models over the other three.

**Figure 4:**
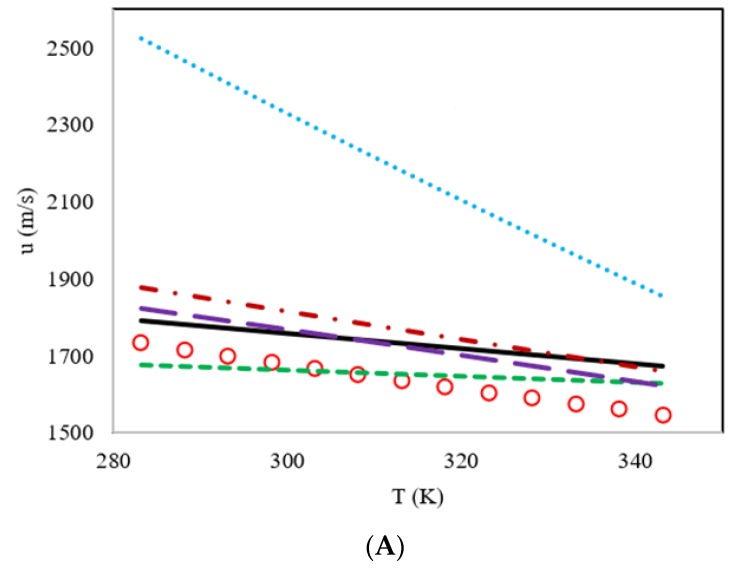

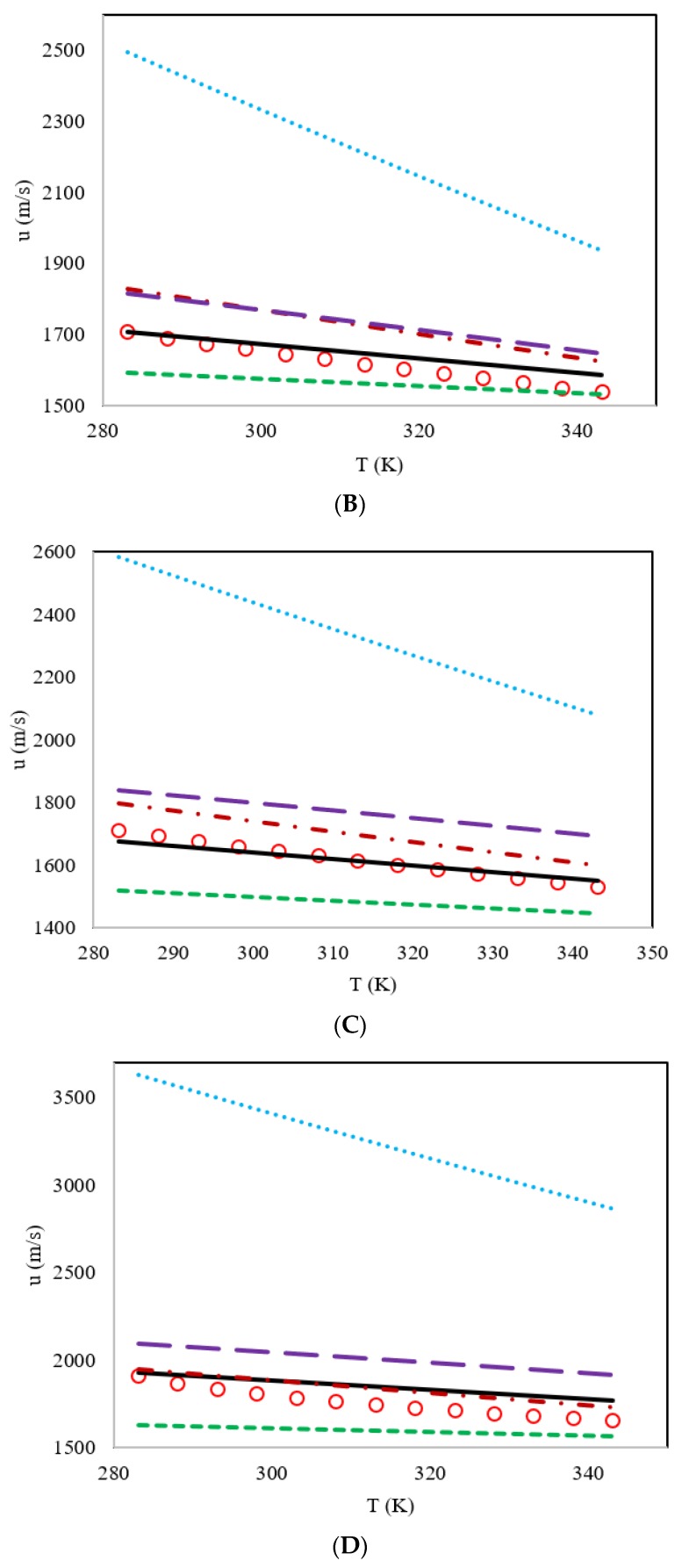
Comparison of the behavior of the speed of sound versus the temperature for the proposed model and literature models for four members of the DES family having benzyl tributyl ammonium chloride as the HBA and the different HBDs of ethylene glycol (DES4) (**A**), diethylene glycol (DES5) (**B**), triethylene glycol (DES6) (**C**) and glycerol (DES7) (**D**). Experimental data **o**, proposed model **―**, Haghbakhsh et al.’s model [24] **---**, Gardas and Coutinho’s model [27] ― ―, Hekayati and Esmaeilzadeh’s model [28] · – · – and Singh and Singh’s model [29] ···.

**Figure 5:**
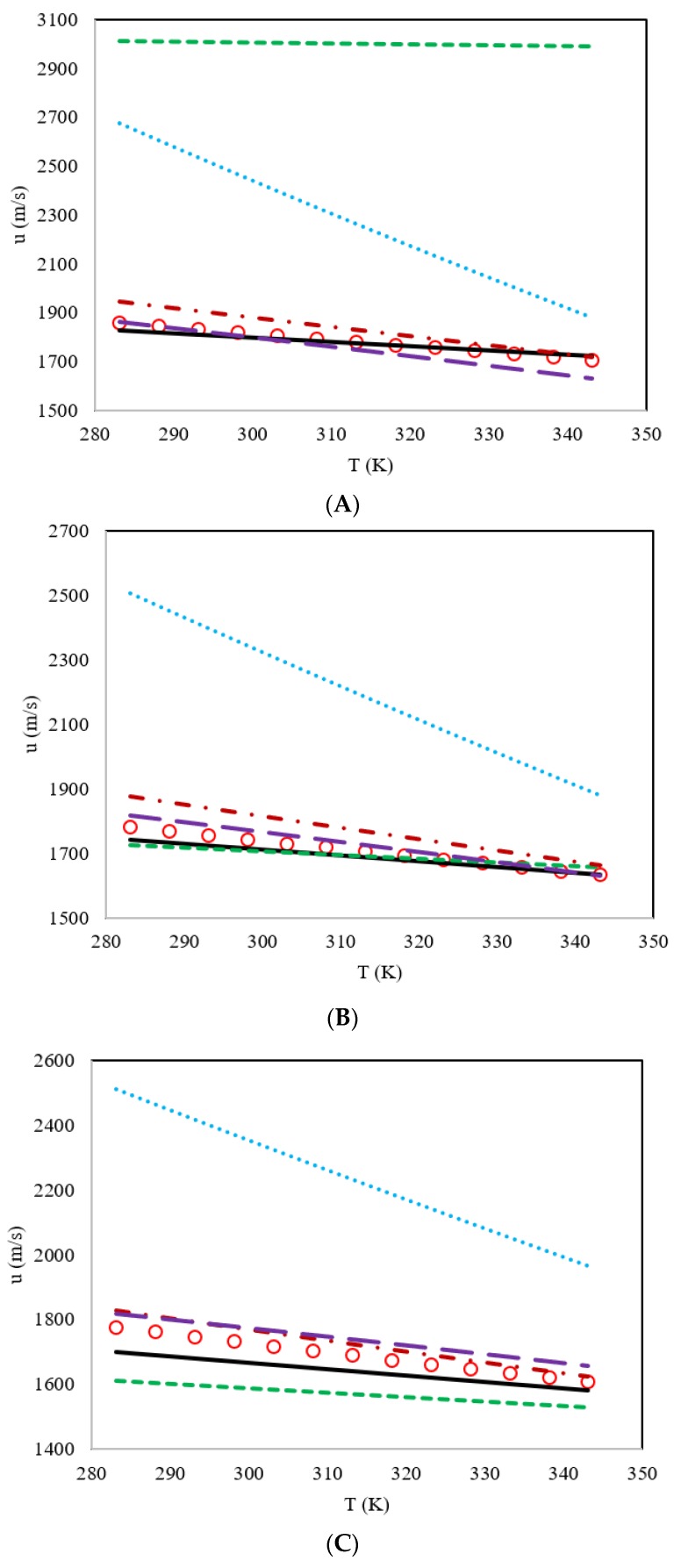

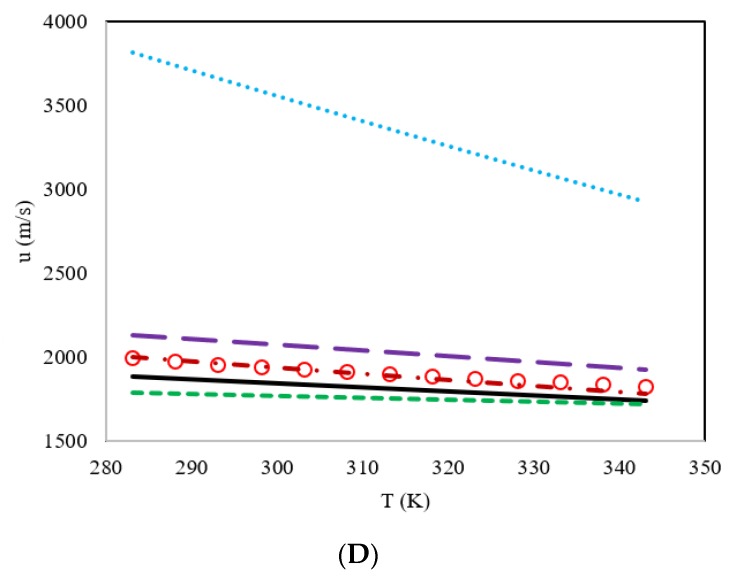
Comparison of the behavior of the speed of sound versus the temperature for the proposed model and literature models for four members of the DES family having benzyl trimethyl ammonium chloride as the HBA and the different HBDs of ethylene glycol(DES8) (**A**), diethylene glycol (DES9) (**B**), triethylene glycol (DES10) (**C**) and glycerol (DES11) (**D**). Experimental data **o**, proposed model **―**, Haghbakhsh et al.’s model [24] **---**, Gardas and Coutinho’s model [27] **― ―**, Hekayati and Esmaeilzadeh’s model [28] **· –** · **–** and Singh and Singh’s model [29] **···**.

In addition to the comparisons of AARD%, the behavior of the speed of sound versus the temperature of the proposed model and the literature models are shown on Figure 4 for DES4, DES5, DES6 and DES7, and on Figure 5 for DES8, DES9, DES10 and DES11. Based on the results shown on Figure 4; Figure 5, it is obvious that all the studied models did indeed estimate a negative slope for the speed of sound versus temperature. However, the model of Singh and Singh [29] shows slopes that are much steeper than the experimental data, while the slopes of the other four models do not differ greatly from one another.

According to Figure 4 for the family of benzyl tributyl ammonium chloride as the HBA, it was concluded that the models of Hekayati and Esmaeilzadeh and Gardas and Coutinho had similar trends, and in most cases, they overestimate, while the model of Haghbakhsh et al. shows the least slopes and mostly underestimates the experimental data and the slopes. In Figure 5, which shows similar graphs, but for the family of benzyl trimethyl ammonium chloride as the HBA, again the models of Hekayati and Esmaeilzadeh and Gardas and Coutinho had almost the same slopes in most cases, and the model of Haghbakhsh et al. underestimates the data and the slopes in most cases, except for the case of ethylene glycol as the HBD, which has completely erroneous results.

The behavior of the speed of sound versus the temperature of the other DESs not presented in Figure 4; Figure 5 are presented in Figures S1–S4 of the Supplementary file. Based on all these results, it is clear that the proposed model was generally the most precise model, yet it is a simple and user-friendly model that can be utilized to estimate the speed of sound of different DESs.

## 4. Conclusions

In this work, a general correlation was introduced for the first time for estimating the speed of sound in DESs. For this purpose, 28 different DESs were used for establishing a proper model, and 11 other DESs were used for testing the capability of the proposed model. The only input parameters of the proposed model were the molecular weight, critical molar volume and the acentric factor of the DES, where the latter two could themselves be calculated with an appropriate group contribution method. Therefore, this model was essentially needless of any input data, making it very widely applicable in comparison to other literature models. For example, the ionic liquid models of Gardas and Coutinho, Hekayati and Esmaeilzadeh and Singh and Singh all require density and surface tension properties as the input parameters for calculating the speed of sound, which can be a limitation for designer solvents. Since no generalized models were, as of yet, available for DESs, the model of this study was compared to literature models proposed for a close relative, the ionic liquids. The results indicated that the AARD% of the proposed model was only 5.4%, while those for the models of Gardas and Coutinho, Hekayati and Esmaeilzadeh, Singh and Singh and Haghbakhsh et al. were 9.1%, 8.8%, 40.8% and 9.7%, respectively. Furthermore, such a low value of AARD%, obtained for 39 different DES types having different HBAs and HBDs, indicates that the proposed model is general and can be applied for estimating the speed of sound of various types of DESs accurately.

## Figures and Tables

**Table 1:** The list of investigated deep eutectic solvents (DESs) in this study and the corresponding hydrogen bond acceptor (HBA), hydrogen bond donor (HBD) and molar ratio of each.

DES #	Data Set	HBA	HBD	HBA:HBD Molar Ratio	Ndp ^1^	Ref.
DES1	Test	1-Ethyl-3-methylimidazolium chloride	Ethylene glycol	2:1	5	[30]
DES2	Training	1-Ethyl-3-methylimidazolium chloride	Ethylene glycol	1:1	5	[30]
DES3	Test	1-Ethyl-3-methylimidazolium chloride	Ethylene glycol	1:2	5	[30]
DES4	Training	Benzyl-tributyl-ammonium-chloride	Ethylene glycol	1:3	13	[31]
DES5	Training	Benzyl-tributyl-ammonium-chloride	Diethylene glycol	1:3	13	[31]
DES6	Training	Benzyl-tributyl-ammonium-chloride	Triethylene glycol	1:3	13	[31]
DES7	Test	Benzyl-tributyl-ammonium-chloride	Glycerol	1:3	13	[31]
DES8	Training	Benzyl-trimethyl-ammonium-chloride	Ethylene glycol	1:3	13	[31]
DES9	Training	Benzyl-trimethyl-ammonium-chloride	Diethylene glycol	1:3	13	[31]
DES10	Test	Benzyl-trimethyl-ammonium-chloride	Triethylene glycol	1:3	13	[31]
DES11	Training	Benzyl-trimethyl-ammonium-chloride	Glycerol	1:3	13	[31]
DES12	Training	Benzyl-tripropyl-ammonium-chloride	Phenol	1:3	11	[32]
DES13	Training	Benzyl-tripropyl-ammonium-chloride	Ethylene glycol	1:3	11	[32]
DES14	Training	Benzyl-tripropyl-ammonium-chloride	Lactic acid	1:3	11	[32]
DES15	Training	Benzyl-tripropyl-ammonium-chloride	Glycerol	1:3	11	[32]
DES16	Training	Betaine	Lactic acid	1:2	10	[21]
DES17	Training	Betaine	Lactic acid	1:5	11	[21]
DES18	Training	Betaine	Levulinic acid	1:2	11	[21]
DES19	Training	Betaine	Lactic acid/water	1:1:1	11	[21]
DES20	Test	Betaine	Citric acid/water	2:1:6	11	[21]
DES21	Training	Choline-Chloride	Urea	1:2	20	[33,34,35]
DES22	Test	Choline-Chloride	Ethylene glycol	1:2	13	[33,34]
DES23	Test	Choline-Chloride	Glycerol	1:2	38	[33,34,36]
DES24	Training	Choline-Chloride	Fructose	2:1	7	[34]
DES25	Test	Choline-Chloride	Glucose	2:1	7	[34]
DES26	Training	Choline-Chloride	1,2propanediol	1:3	10	[37]
DES27	Training	Choline-Chloride	Levulinic acid	1:2	11	[38]
DES28	Training	Choline-Chloride	Malonic acid	1:1	7	[39]
DES29	Test	Choline-Chloride	Glutaric acid	1:1	7	[39]
DES30	Training	Choline-Chloride	Oxalic acid	1:1	4	[40]
DES31	Training	Dodecanoic acid	Octanoic acid	1:3	11	[41]
DES32	Training	Dodecanoic acid	Decanoic acid	1:2	10	[41]
DES33	Training	Menthol	Octanoic acid	1:1	11	[41]
DES34	Training	Menthol	Decanoic acid	1:1	11	[41]
DES35	Training	Menthol	Salicylic acid	4:1	5	[42]
DES36	Test	Menthol	Camphor-10-sulfonic acid	5:1	5	[42]
DES37	Training	Menthol	Ethylene glycol	1:1	5	[42]
DES38	Test	Proline	Levulinic acid	1:2	11	[21]
DES39	Training	Proline	Lactic acid	1:1	10	[21]
**Total**					**420**	

^1^ Number of data points.

**Table 2:** Calculated values of critical properties and acentric factors for all of the investigated DESs in this study [22,47].

DES #	Tc (K)	Pc (bar)	Vc (cm^3^/mol)	ω	Mw (g/mol)
DES1	670.98	36.65	355.99	0.6660	118.44
DES2	651.23	39.77	308.96	0.7476	104.34
DES3	632.35	43.77	264.25	0.8293	90.25
DES4	657.28	31.24	364.48	0.9659	124.53
DES5	720.58	25.62	480.62	0.9994	157.57
DES6	778.21	22.07	589.83	1.0507	190.61
DES7	749.11	25.67	433.69	1.3146	147.05
DES8	618.43	41.08	270.56	0.8745	92.97
DES9	678.15	31.88	377.22	0.9080	126.01
DES10	733.31	26.60	478.88	0.9593	159.05
DES11	708.07	32.89	333.89	1.2232	115.49
DES12	701.16	37.82	380.25	0.5152	138.05
DES13	644.10	33.78	334.18	0.9375	114.02
DES14	721.27	33.15	384.56	0.9166	135.02
DES15	735.27	27.58	401.61	1.2862	136.53
DES16	668.50	44.09	281.96	0.7863	99.10
DES17	683.07	47.23	259.82	0.8755	94.59
DES18	701.24	38.94	356.12	0.6195	116.46
DES19	637.98	61.84	206.94	0.5794	75.08
DES20	659.71	92.43	146.46	0.5139	59.39
DES21	644.44	49.54	254.37	0.6509	86.58
DES22	602.00	40.99	259.67	0.9155	87.92
DES23	680.67	33.46	315.17	1.2254	107.94
DES24	742.22	27.03	424.87	1.2278	153.13
DES25	738.99	27.23	422.14	1.2163	153.13
DES26	620.93	38.44	284.11	0.9290	91.98
DES27	702.19	35.40	376.78	0.7301	123.95
DES28	689.82	37.16	335.84	0.8577	121.84
DES29	713.43	32.24	397.17	0.8782	135.87
DES30	676.24	40.44	303.06	0.8531	114.83
DES31	737.07	24.71	559.27	0.7649	158.24
DES32	773.88	21.55	656.40	0.8307	181.61
DES33	717.72	28.79	493.39	0.6173	150.24
DES34	739.17	26.26	549.11	0.6568	164.27
DES35	744.23	33.56	445.77	0.5733	152.64
DES36	777.87	31.66	504.89	0.5094	168.94
DES37	654.33	38.54	319.91	0.7510	109.17
DES38	745.61	42.88	333.41	0.7044	115.78
DES39	721.95	48.54	272.60	0.8243	102.61

**Table 3:** The number of investigated data in the different datasets and the corresponding AARD% of the proposed model.

Data Set	Number of Investigated Data	AARD%
Training	292	4.8
Test	128	6.8
Overall	420	5.4

**Table 4:** Comparison of the values of AARD% for the proposed model and literature models for each of the investigated DESs.

DES	Proposed Model	Haghbakhsh et al.’s Model [24]	Hekayati and Esmaeilzadeh’s Model [28]	Gardas and Coutinho’s Model [27]	Singh and Singh’s Model [29]
DES1	13.9	14.5	8.6	15.0	4.9
DES2	7.5	16.6	3.6	9.0	11.8
DES3	1.3	17.6	2.6	1.3	31.6
DES4	5.8	2.4	8.1	5.2	33.0
DES5	1.8	3.3	6.6	7.1	36.5
DES6	0.9	8.3	5.0	9.3	43.8
DES7	5.4	9.0	4.7	14.2	84.5
DES8	0.7	68.8	2.8	1.9	27.2
DES9	1.1	1.4	3.6	1.1	28.0
DES10	2.8	7.0	2.1	2.9	32.1
DES11	4.6	7.6	0.9	6.8	76.5
DES12	1.9	2.7	3.6	3.6	4.4
DES13	2.5	1.8	4.8	1.2	26.1
DES14	8.9	22.0	11.9	13.7	54.0
DES15	1.3	9.0	2.1	10.9	77.4
DES16	1.6	1.3	4.6	2.8	37.4
DES17	12.5	7.6	16.7	20.1	82.7
DES18	2.8	2.0	4.9	1.0	15.8
DES19	1.1	1.4	0.8	3.8	29.6
DES20	2.4	1.5	1.8	6.2	79.5
DES21	12.6	25.2	10.4	15.5	7.8
DES22	4.7	10.4	2.6	7.9	13.8
DES23	8.1	5.2	4.5	4.1	66.2
DES24	1.0	4.7	4.6	3.6	55.2
DES25	21.4	25.5	25.7	19.5	19.1
DES26	2.5	11.6	8.8	4.0	36.6
DES27	4.5	4.6	2.7	1.3	18.9
DES28	5.9	3.6	3.7	4.2	30.9
DES29	8.7	1.3	4.0	4.5	26.4
DES30	3.9	6.8	2.6	2.7	39.3
DES31	3.3	15.4	31.7	25.5	36.0
DES32	7.1	6.6	27.3	25.3	39.1
DES33	7.7	13.1	29.0	19.5	22.7
DES34	2.1	11.6	26.1	18.9	23.5
DES35	9.2	3.1	24.3	17.8	36.8
DES36	2.6	5.7	19.7	13.3	26.7
DES37	21.2	2.6	28.0	20.8	53.8
DES38	6.0	2.2	12.8	13.7	56.8
DES39	4.8	2.6	7.8	13.1	75.8
**Total**	**5.4**	**9.7**	**8.8**	**9.1**	**40.8**

## Supplementary Materials

The following are available online: Figure S1: Comparison of the behavior of speed of sound versus temperature of the proposed model and literature models for the three members of a DES family including 1-Ethyl-3-methylimidazolium chloride as HBA and different molar ratios of ethylene glycol as HBD. (DES3) (A), (DES2) (B) and (DES1) (C), Figure S2: Comparison of the behavior of the speed of sound versus temperature of the proposed model and literature models for the four members of a DES family including Benzyl tripropyl ammonium chloride as HBA and different HBDs of phenol (DES12) (A), ethylene glycol (DES13) (B), lactic acid (DES14) (C) and glycerol (DES15) (D), Figure S3: Comparison of the behavior of speed of sound versus temperature of the proposed model and literature models for the five members of a DES family including Betaine as HBA and different HBDs. DES16 (A), DES17 (B), DES18 (C), DES19 (D) and DES20 (E), Figure S4: Comparison of the behavior of speed of sound versus temperature of the proposed model and literature models for the ten members of a DES family including Choline chloride as HBA and different HBDs. DES21 (A), DES22 (B), DES23 (C), DES24 (D), DES25 (E), DES26 (F), DES27 (G), DES28 (H), DES29 (I) and DES30 (J).
