# Supplementary material for: A Global Model for the Estimation of Speeds of Sound in Deep Eutectic Solvents
Source: Molecules. 2020 Apr 1;25(7):1626. doi: 10.3390/molecules25071626 (PMC7180493; doi:10.3390/molecules25071626)
Supplement: Supplementary file 1 [file molecules-25-01626-s001.pdf]

Supplementary Information

A Global Model for Estimation of Speeds of Sound in Deep Eutectic Solvents

Hamed Peyrovedin [1], Reza Haghbakhsh [1,2], Ana Rita C. Duarte [2] and Sona Raeissi [1,*]

[1] School of Chemical and Petroleum Engineering, Shiraz University, Mollasadra Ave., Shiraz 71348-51154, Iran.
[2] LAQV, REQUIMTE, Departamento de Química da Faculdade de Ciências e Tecnologia, Universidade Nova de Lisboa, 2829-516 Caparica, Portugal.
* Correspondence: raeissi@shirazu.ac.ir; +98-71-36133707

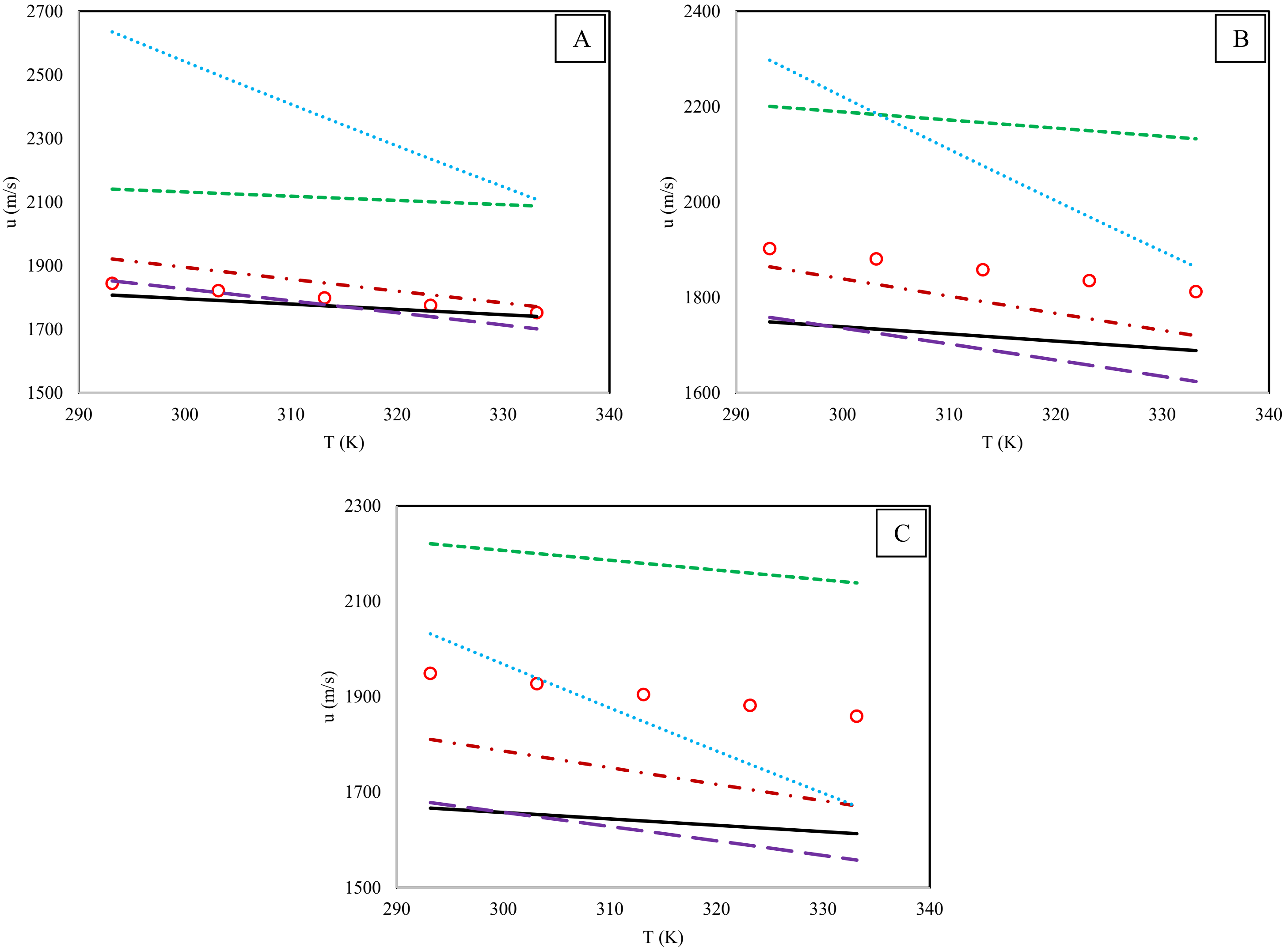

Figure S1. Comparison the behavior of speed of sound versus temperature of the proposed model and literature models for the three members of a DES family including 1-Ethyl-3-methylimidazolium chloride as HBA and different molar ratios of ethylene glycol as HBD. (DES3) (A), (DES2) (B) and (DES1) (C). (Experimental data **o**, proposed model —, Haghbakhsh et al. model [1] ---, Gardas and Coutinho model [2]— —, Hekayati and Esmaeilzadeh model [3] · — · —, Singh and Singh model [4] ···)

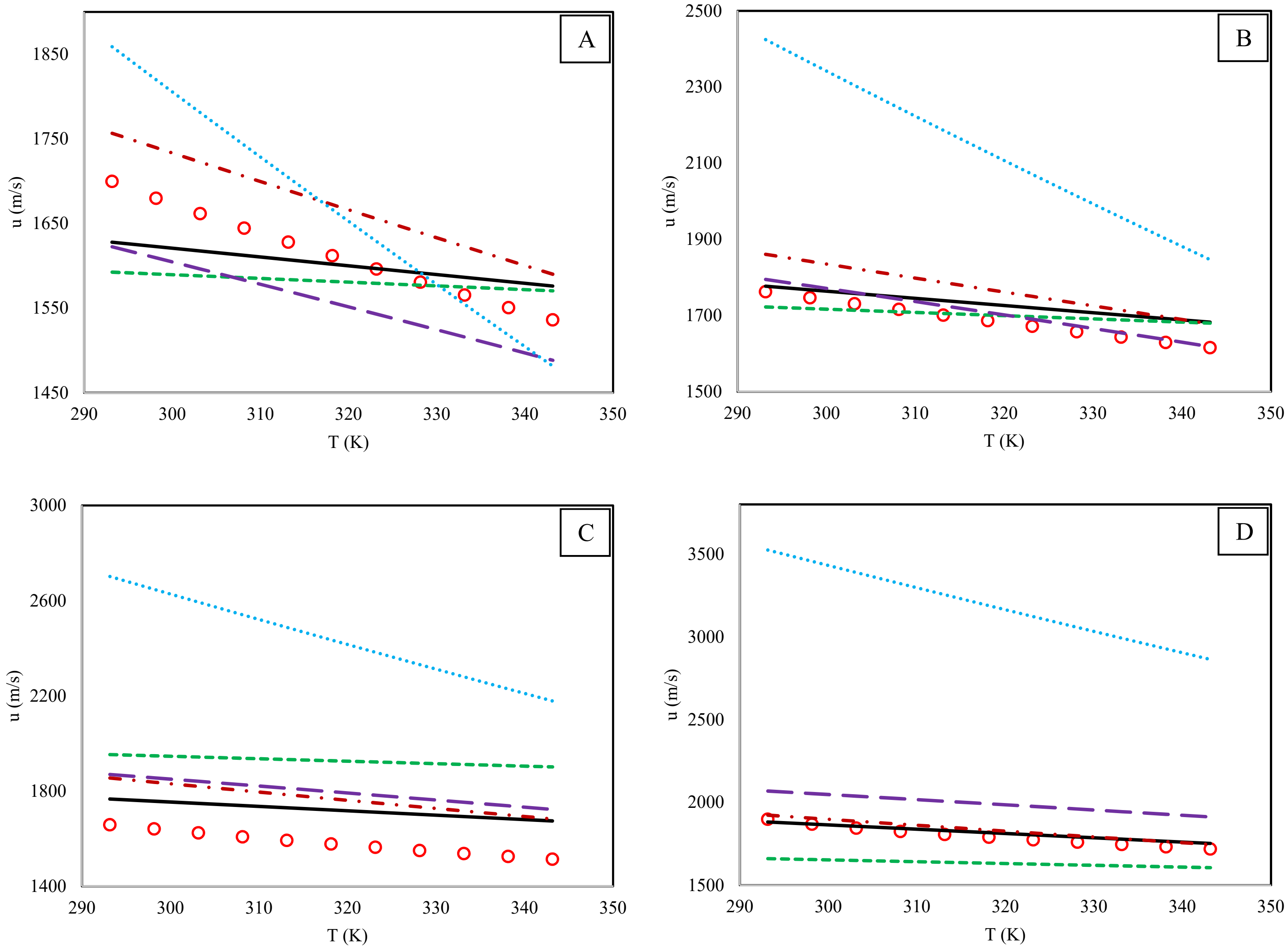

Figure S2. Comparison the behavior of speed of sound versus temperature of the proposed model and literature models for the four members of a DES family including Benzyl tripropyl ammonium chloride as HBA and different HBDs of phenol (DES12) (A), ethylene glycol (DES13) (B), lactic acid (DES14) (C) and glycerol (DES15) (D). (Experimental data **o**, proposed model —— , Haghbakhsh et al. model [1] - - -, Gardas and Coutinho model [2]— —, Hekayati and Esmaeilzadeh model [3] • – • –, Singh and Singh model [4] •••

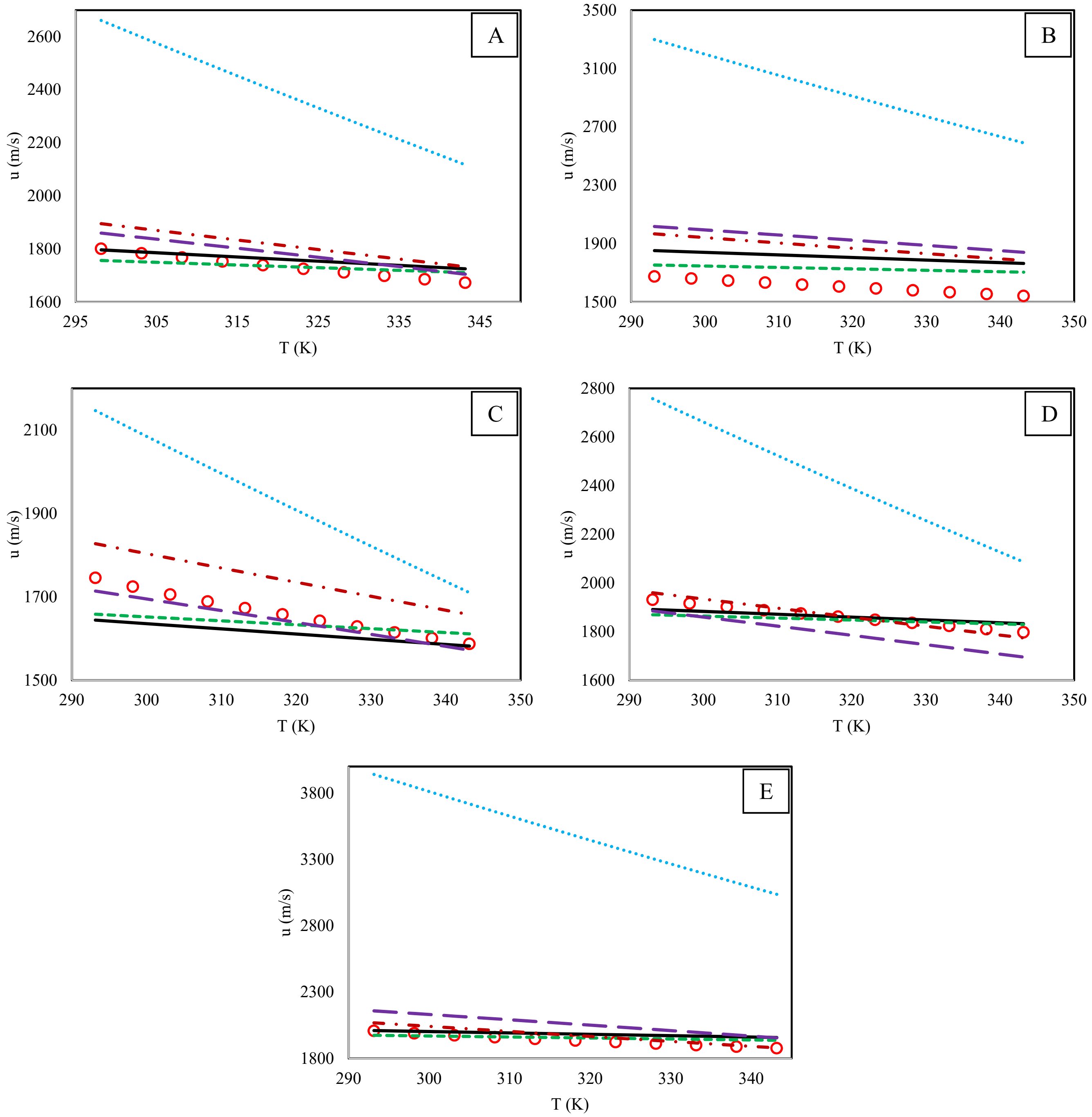

Figure S3. Comparison the behavior of speed of sound versus temperature of the proposed model and literature models for the five members of a DES family including Betaine as HBA and different HBDs. DES16 (A), DES17 (B), DES18 (C), DES19 (D) and DES20 (E). (Experimental data **o**, proposed model —, Haghbakhsh et al. model [1] ---, Gardas and Coutinho model [2]— —, Hekayati and Esmaeilzadeh model [3] · – · –, Singh and Singh model [4] ···)

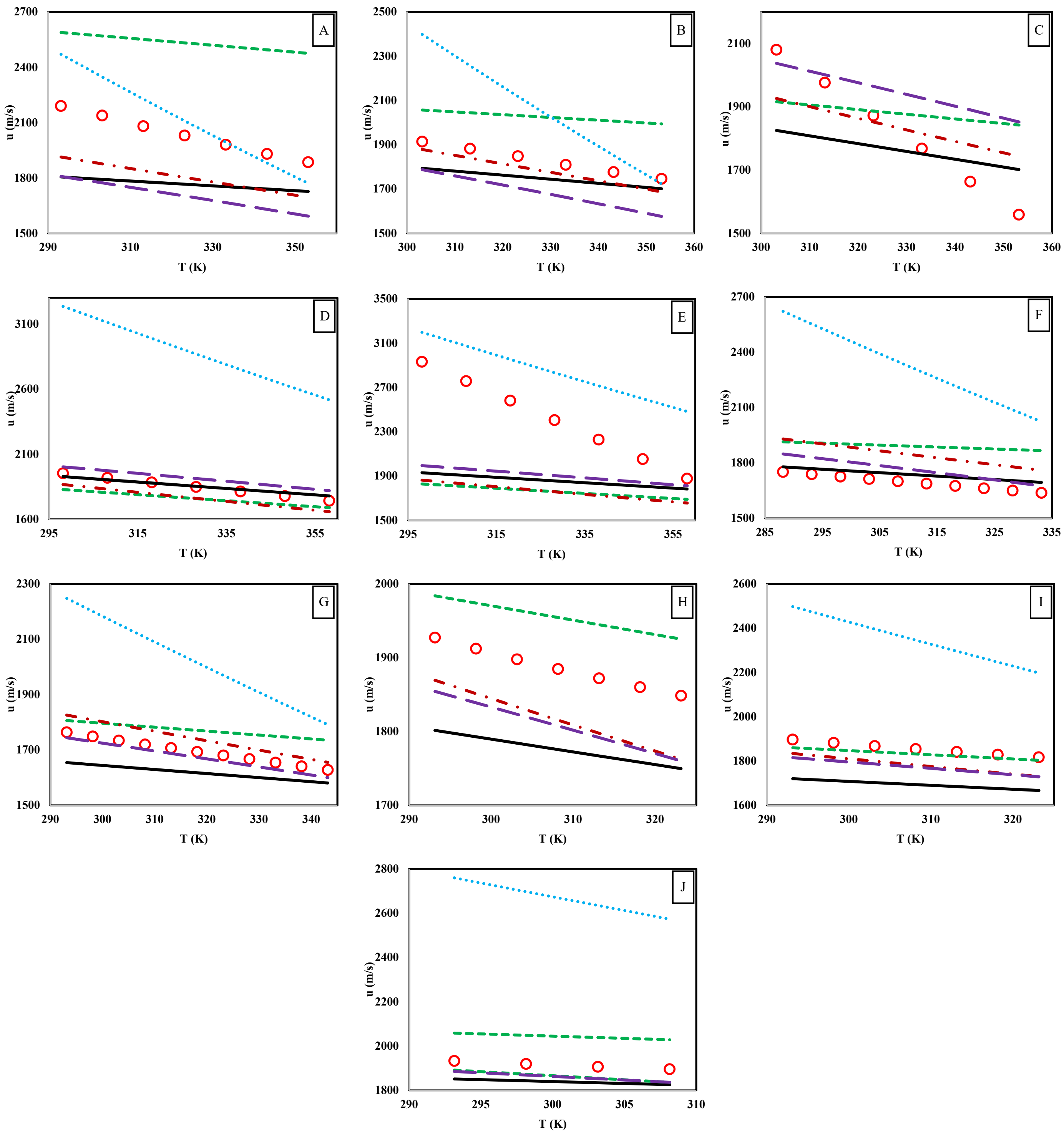

Figure S4. Comparison the behavior of speed of sound versus temperature of the proposed model and literature models for the ten members of a DES family including Choline chloride as HBA and different HBDs. DES21 (A), DES22 (B), DES23 (C), DES24 (D), DES25 (E), DES26 (F), DES27 (G), DES28 (H), DES29 (I) and DES30 (J). (Experimental data **o**, proposed model —, Haghbakhsh et al. model [1] ---, Gardas and Coutinho model [2]— —, Hekayati and Esmaeilzadeh model [3] •—•—, Singh and Singh model [4] •••)

References

[1] Haghbakhsh, R.; Keshtkari, S.; Raeissi, S. Simple estimations of the speed of sound in ionic liquids, with and without any physical property data available. *Fluid Phase Equilib.* **2020**, *503*, doi:https://doi.org/10.1016/j.fluid.2019.112291.

[2] Gardas, R.L.; Coutinho, J.A.P. Estimation of speed of sound of ionic liquids using surface tensions and densities: A volume based approach. *Fluid Phase Equilib.* **2008**, *267*, 188-192, doi:https://doi.org/10.1016/j.fluid.2008.03.008.

[3] Hekayati, J.; Esmaeilzadeh, F. Predictive correlation between surface tension, density, and speed of sound of ionic liquids: Auerbach model revisited. *J. Mol. Liq.* **2019**, *274*, 193-203, doi:https://doi.org/10.1016/j.molliq.2018.10.099.

[4] Singh, M.P.; Singh, R.K. Correlation between ultrasonic velocity, surface tension, density and viscosity of ionic liquids. *Fluid Phase Equilib.* **2011**, *304*, 1-6, doi:https://doi.org/10.1016/j.fluid.2011.01.029.
